# Supplementary material for: Phototroph-heterotroph interactions during growth and long-term starvation across Prochlorococcus and Alteromonas diversity
Source: ISME J. 2022 Nov 5;17(2):227–37. doi: 10.1038/s41396-022-01330-8 (PMC9860064; doi:10.1038/s41396-022-01330-8)
Supplement: Supplementary file 1 — Supplemental material [file 41396_2022_1330_MOESM1_ESM.pdf]

Supplementary Data

Phototroph-heterotroph interactions during growth and long-term starvation across *Prochlorococcus* and *Alteromonas* diversity

Osnat Weissberg, Dikla Aharonovich and Daniel Sher

Department of Marine Biology, Leon H. Charney School of Marine Sciences, University of Haifa, Israel

Supplementary text S1 Differences between the current study and previous systematic analyses of *Prochlorococcus*-heterotroph interactions

In a previous study (Sher et al. 2011), the interactions of multiple heterotrophic bacteria with two *Prochlorococcus* strains were studied in high throughput [1]. This included also multiple *Alteromonas* strains, which inhibited *Prochlorococcus* MIT9313 (resulting in a “late growth” phenotype) and had little discernable effect on *Prochlorococcus* MED4. How does the current study build upon the Sher et al. 2011 study, what are the differences in experimental design, and how could these affect the interpretations?

1) Sher et al. 2011 paper focused on the initial phenotype when previously-axenic strains encounter each other (i.e. similar to experiment E1). A question emerged, therefore, whether these phenotypes could change over time, e.g. as the two cell types acclimate to the presence of each other. Here we study the interactions across multiple cycles of growth and nitrogen starvation.
2) The limiting factor for *Prochlorococcus* growth was not defined or known in the Sher et al. 2011 paper, where the cultures were grown in Pro99 media with a limited amount of added organic matter ([2] and see below). Here, we aimed to characterize the response to a known limiting nutrient, in this case N, leading to a clearly defined process of growth cessation due to N starvation [3].
3) The interaction stages and culture life cycle studied are different between the two experiments. In the Sher et al. 2011 paper we focused on growth, including the extraction of relevant parameters (e.g. Figure 2 in [1]). Here we focus on the decline stage, and on quantifying mortality.

4) In the Sher et al. 2011 paper the experiments were performed in high throughput, requiring the transfer of a known volume of heterotroph culture into a *Prochlorococcus* culture in a 96 well plate (2 μl into 200 μl). This led to the transfer, with the heterotroph cells, of a limited (and unknown) amount of organic matter from the heterotroph growth media. Under these conditions, heterotrophic bacteria initially grow without the need for organic C from *Prochlorococcus*, leading to a lack of relationship between initial heterotroph density and co-culture phenotype (Supplementary text and Supplementary Figure S2 in [1]). Furthermore, in the Sher et al. 2011 paper the initial heterotroph concentrations were not controlled or measured. In contrast, essentially all of the organic matter supporting the growth of the heterotrophs in the present study is supplied by *Prochlorococcus*, leading to a much higher dependence of the heterotroph on *Prochlorococcus* (and a dependence of the interaction phenotype on the "dose" of heterotrophs, see [4]). These differences in experimental setup enable the analysis of carrying capacity presented in this study.
5) The diversity of heterotrophic partners was higher in the Sher et al. 2011 paper, whereas the current paper includes a higher diversity of *Prochlorococcus* strains and ecotypes. Specifically, for the *Alteromonas* strains, the current study includes strains belonging to two species (*A. macleodii* and *A. Mediterranea*), one of which (*Alteromonas* HOT1A3) was first described in the Sher et al. 2011 paper. In contrast, the Sher et al. 2011 includes primarily *A. macleodii*, with several members putatively associated with *A. lipolytica*.

Supplementary text S2 **The differences between *Prochlorococcus* strains are most evident in the decline and long-term starvation stages**

We used random forest classification to test whether the observation that the co-culture outcome is determined by the *Prochlorococcus* and not *Alteromonas* strains, suggested by the clustering of the growth curves observed in the PCA ordination, is reproduced using an independent method. We then quantitatively determined which stages of the co-culture were most different between the *Prochlorococcus-Alteromonas* pairs. Random forest [5] is a supervised machine learning algorithm widely used in classification and regression problems because it produces relatively accurate results while avoiding overfitting. The forest model is built on a large number of

individual decision trees. Each individual tree computes class prediction and the model predicts the class with most votes. Random forest thus relies on 'crowd sourcing' to the individual trees in order to avoid overfitting. Random forest has the added benefit that it provides feature importance, a measure of how much the model relies on each feature for classification. We used 10 x cross validation in which the ability of the algorithm to accurately predict outcome is tested by building the model on 90% of the samples and testing its accuracy on the 10% of samples that were not used in the model building. This process is repeated 10 times for different sets of samples. The algorithm accurately predicted, from the growth curves, the *Prochlorococcus* strain (accuracy 0.92±0.17) but performed much more poorly when trying to predict the *Alteromonas* strain (accuracy 0.62 ± 0.58) (Supplementary Figure S2A). Providing further support for the observation that the shape of the growth and decline curves was driven by the specific *Prochlorococcus* strain and not by the *Alteromonas*. The classification of the *Prochlorococcus* strains relied heavily on days during the decline phase where the dynamics of decline differed between strains. For example, around day 40 the decline rate of MIT9313 (LLIV) co-cultures seems to increase, whereas those of MIT9312 (HLII) were stable and the fluorescence of NATL2A (LLI) co-cultures actually increased (Supplementary Figure S2B).

Supplementary text S3: The phenotype of interactions is maintained over multiple cycles of growth-decline-starvation

In all the subsequent transfers, the co-cultures declined relatively slowly, and were able to survive transfer for up to 140 days (Figure 1). The different *Prochlorococcus* strains could still be differentiated in a PCA ordination based on their growth curves (Adonis, R^2 = 0.37-0.47, p=0.001; Supplementary Figure S3), although the difference between low-light adapted NATL2A strain and the high-light adapted strains was less pronounced. Similar to E1, the clustering by *Alteromonas* strain was not as significant and r values were lower (Adonis, R^2 = 0.05-0.16, see p in Figure S3 legend). These results are repeated in random rorest classification. The accuracy of *Prochlorococcus* strain classification on subsequent transfers is 0.8-0.9, while the accuracy of *Alteromonas* classification is only 0.2-0.65 (Supplementary Figure S2A). Thus, the differences between *Prochlorococcus* strains in the way in which they interact with multiple *Alteromonas*

strains (and the lack of any observed effect of the *Alteromonas* strains) are robust to the initial cell numbers and the time in co-culture.

Nevertheless, there were some consistent changes between E1 and all subsequent experiments. Firstly, the maximum fluorescence decreased in subsequent co-cultures compared to E1 (ANOVA, $p<0.05$, Supplementary Figure S4A). Secondly, the growth rate of *Prochlorococcus* in co-culture increased in most strains and experiments (ANOVA, $p<0.05$, Supplementary Figure S4C). Thirdly, MIT9313 (LLIV), which was inhibited in the first co-culture by all *Alteromonas* strains, did not show this phenotype in subsequent transfers (Figure 1B, Supplementary Figure S4B). This is potentially due to the lower number of inoculated *Alteromonas* cells, as shown previously [4].

Supplementary text S4: Carrying Capacity of the cultures

The carrying capacity of the cultures was defined as the amount of nitrogen retained in cell biomass (rather than as dissolved organic N) at various stages of long-term co-culture. Cell numbers from flow cytometry were converted into nitrogen using 7 fg N $cell^{-1}$ for the high-light strains MED4, MIT9312, and MIT0605, 10.5 fg N $cell^{-1}$ for strain NATL2A and 14 fg N $cell^{-1}$ for strain MIT9313 [6, 7]. For *Alteromonas* we used a value of 13 fg N $cell^{-1}$ [8, 9]. We note that the values we used are at the lower end of measured cell values, which reach up to 20 fg N $cell^{-1}$ for low-light *Prochlorococcus* and 25 fg N $cell^{-1}$ for *Alteromonas*, since using the higher N cell quota leads to biomass that is higher than the total nitrogen available in the system. This assumption is supported by studies showing that cells contain less nitrogen under long term N stress compared to exponential growth [10–12].

The cell numbers were converted to µmol/L by the formula:

biomass [µmol/L] = X [cell/ml] * Q_N^{PRO} [fg/cell] * 1e-9 [converting femtomol-> micromol] / 1e-3 [ml->L] / MW_N [g/mol]

Where X is the number of cells per ml, Q_N is the cell N quota, and MW_N is the molecular weight of nitrogen.

Since neither *Prochlorococcus* nor *Alteromonas* are known to fix nitrogen or perform denitrification, we assume that the co-cultures are closed systems for N (i.e. the total N in the system does not change over time). The experiment media contains 100 µmol / L of NH_4 (dissolved

inorganic nitrogen, DIN), an estimated 5 µmol/L of dissolved organic nitrogen (DON) [13] from the natural seawater used for the media, and the N biomass of the inoculated cells. The initial N in the media is calculated as:

initial_N = 100 [DIN] + 5 [DON] +

Q_N^{PRO} [fg/cell] * 1e6 [cell/ml] * 1e-6 [f->u, ml->L] / 14 [MW_N] +

Q_N^{ALT} [fg/cell] * 1e7 [cell/ml] * 1e-6 [f->u, ml->L] / 14 [MW_N]

Quantifying chlorotic *Prochlorococcus* cells and differentiating between them and (naturally non-pigmented) *Alteromonas* is non-trivial, as these can partially overlap in the flow cytometry scattergrams [3, 12]. While, in theory, genetic measurements such as 16S sequencing or fluorescent in situ hybridization (FISH) could differentiate between these cases, such measurements are not available. Nevertheless, while we cannot rule out that some of the cells counted as *Alteromonas* in the co-cultures are in fact (chlorotic-dead) *Prochlorococcus*, this does not qualitatively affect our results. To demonstrate this, if we assume that 50% of the *Alteromonas* in the co-cultures are in fact chlorotic *Prochlorococcus* (a highly unlikely result) the total N biomass of the co-cultures on day 60 is still ~30% higher than the axenic *Alteromonas*, and is ~3 fold higher on day 100. Thus, in both cases, the overall observation of higher carrying capacity in the co-cultures remains valid. In such case the fold change of *Alteromonas* N biomass (co-culture compared to axenic culture) would be lower than shown in Figure 3C, implying more competition compared with synergism, especially on day 60. Finally, if some of the *Prochlorococcus* cells are in fact chlorotic rather than lyzed this does not affect the results of the modelling of mortality, because these cells are non-viable, meaning that they will eventually lyze.

Supplementary text S5: Interpretation of the bi-exponential decline rates of axenic culture and co-cultures

The bi-exponential model represents two separate subpopulations in the community, each with its own death rate [14]. Supplementary Table S3 shows the fitting of the decline stage of the cultures to a bi-exponential model. Three major groups emerge (one co-culture curve was a clear outlier and was removed from this analysis): i) All of the axenic cultures, where the two death rates are identical, meaning that there is only a single population which declines exponentially. ii) A group of 13 co-cultures, which also have identical decline rates (single population), but these

decline rates are about three-fold lower than those of the axenic cultures (student t-test $p = 1.6e\text{-}23$). iii) The majority of the co-cultures, which had two populations, each with its own decline rate. The larger population, representing 71±15% of the cells, declined at a rate slightly higher than the axenic ones (student t-test $p = 0.01$). The second sub-population, however, declined at a much lower rate (~15-fold lower than the axenic cultures). Therefore, the bi-exponential model predicts that, in the presence of *Alteromonas*, about 30% of the *Prochlorococcus* cells form a highly resilient subpopulation with a decline rate that is more than 10-fold lower than the axenic cells or the fast-dying subpopulation (Supplementary Table S3).

Supplementary Tables

Table S1: N carrying capacity

	Available N [µmol N/L]	Total N Biomass [µmol N/L]		
		**Co-culture**	***Alteromonas* Only**	***Prochlorococcus* Only**
**N**		71	13	14
**Day 0**	DIN: 100<br>DON: 5<br>PRO: 0.7<br>ALT.: 18<br>Total: 123.6			
**Day 60**		PRO: 16±11<br>ALT: 54±29<br>Total: 69±35<br>% of available N: 56±28%	32±22<br>% of available N: 26±18%	0.01±0.00<br>% of available N: 0%
**Day 100**		PRO: 6±4<br>ALT: 18±8<br>Total: 23±10<br>% of available N: 19±8%	4±4<br>% of available N: 3±3%	0.03±0.12<br>% of available N: 0%
**Day 140**		PRO: 0.5±0.8<br>ALT: 0.8±0.7<br>Total: 1.3±1.1<br>% of available N: 1±1%	Not measured	0<br>% of available N: 0%

* PRO: *Prochlorococcus*, ALT: *Alteromonas*

Table S2: Decline model data

		E1 Axenic (n=13)	E1 Co-culture (n=73)	E2 Co-culture (n=72)	E3 Co-culture (n=65)	E2.1 Co-culture (n=69)	E2.2 Co-culture (n=64)
**Weibull shape**		2.10±0.88	0.43±0.17	0.48±0.32	0.48±0.40	0.65±0.34	0.53±0.16
**RMSE**	Exponential	0.26±0.11	0.51±0.30	0.21±0.10	0.22±0.12	0.24±0.14	0.23±0.14
	Biexponential	0.26±0.11	0.27±0.17	0.13±0.07	0.12±0.09	0.13±0.06	0.14±0.09
	Harmonic	0.50±0.19	0.32±0.20	0.15±0.08	0.14±0.07	0.16±0.09	0.17±0.09
	Weibull	0.14±0.08	0.29±0.13	0.16±0.09	0.13±0.07	0.15±0.08	0.16±0.08
**BIC**	Exponential	-231±80	-116±82	-231±78	-117±45	-221±87	-142±65
	Biexponential	-222±80	-208±79	-296±92	-157±50	-296±77	-176±65
	Harmonic	-122±81	-187±77	-274±79	-151±48	-275±87	-166±64
	Weibull	-331±97	-188±57	-268±90	-151±45	-279±79	-167±58

Table S3: Decline bi-exponential model

	n	a1	a2	f	BIC	RMSE
**Axenic 1 sub-population**	13	0.24±0.05	N/A	N/A	-228.47±78.08	0.25±0.11
**Co-Culture 1 sub-population**	13	0.09±0.04	N/A	N/A	-142.74±83.89	0.40±0.23
**Co-Culture 2 sub-populations**	59	0.32±0.20	0.02±0.01	0.72±0.15	-221.69±70.27	0.24±0.14
**Co-Culture 2 sub-populations outlier**	1	13.25	0.02	0.23	-222.09	0.24

Supplementary Figures

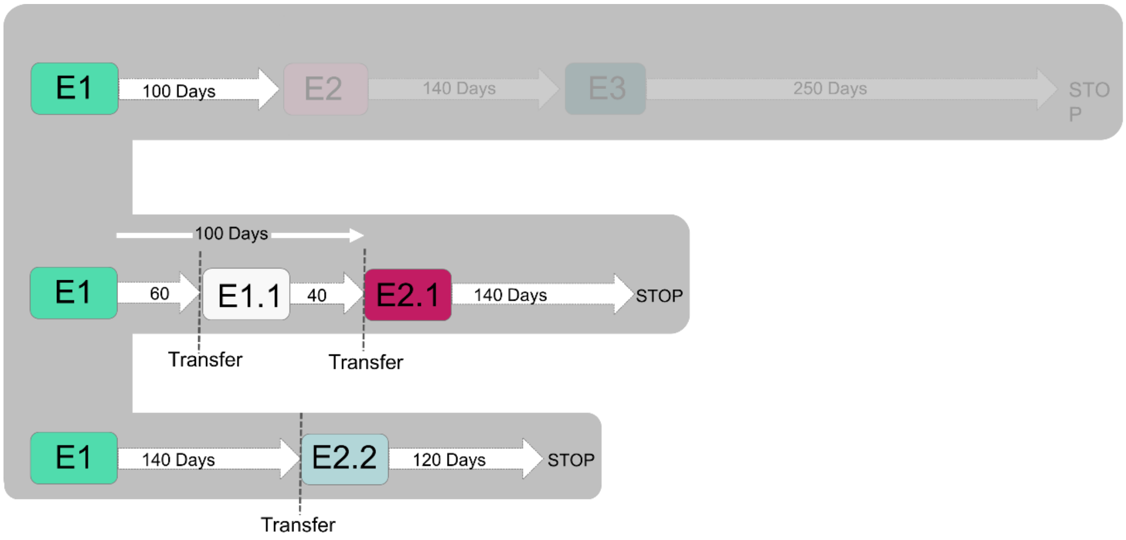

Figure S1: Additional Independent transfers from E1. Three separate transfers were performed from the first experiment (E1). The top line represents experiments E2 and E3, which are the focus of the study and are shown in Figure 1B, C. Two additional transfers were performed, further supporting some of the observations described in the main text. The middle line shows two short transfers from E1 (after 60 and then 40 days, E1.1), to assess the viability of the axenic cultures. These were then transferred subsequently to E2.1, which was maintained for 140 days. The lower line shows an additional transfer from E1 after 140 days to E2.2, which was maintained for another 120 days. E2.1 therefore represents a culture transferred after relatively short N starvation, whereas E2.2 represents an independent replicate of transfer after long-term starvation (similar to E2 but after longer starvation).

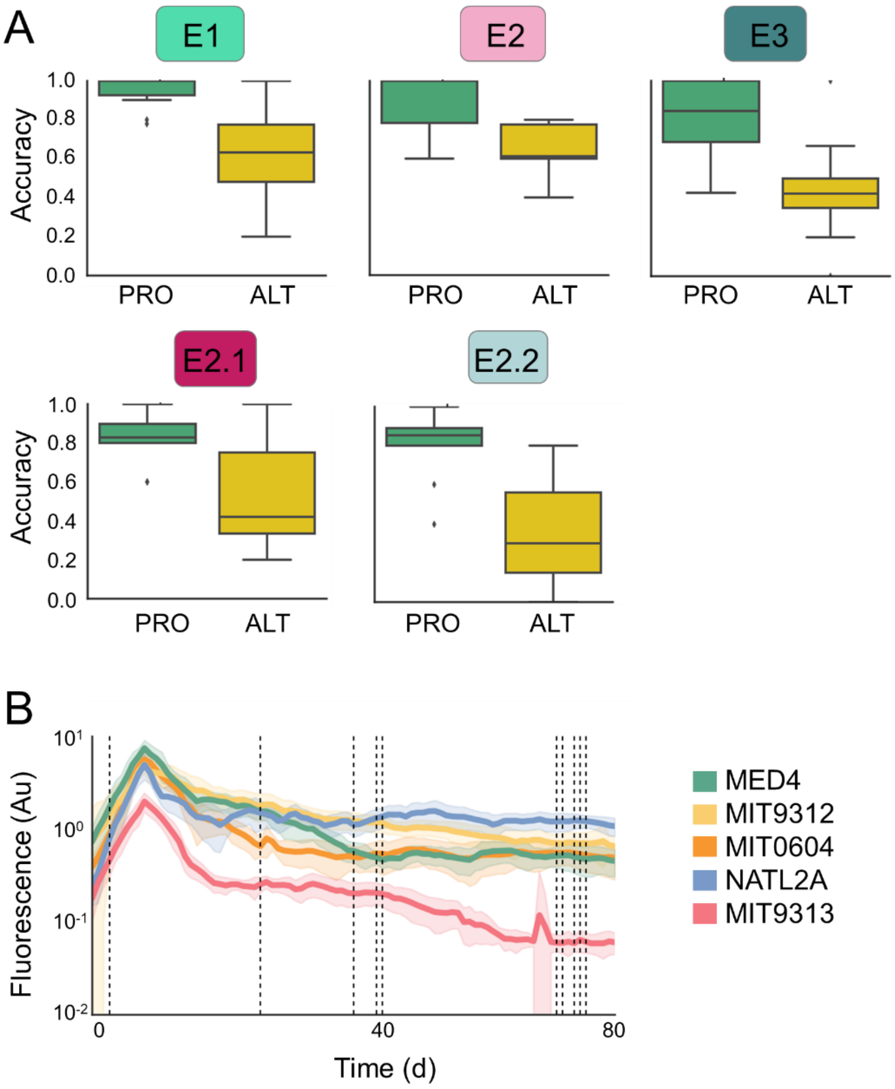

Figure S2: Classification of *Prochlorococcus* and *Alteromonas* strains and identification of important culture stages differentiating between strains using machine learning. A. Random forest accuracy of 10x cross validation when predicting *Prochlorococcus* (PRO) and *Alteromonas* (ALT) classification based on their growth curves. The algorithm has higher cross-validation accuracy in predicting *Prochlorococcus* strain in all experiments/transfers, suggesting that the *Prochlorococcus* strain, and not the *Alteromonas* one*,* has a stronger effect on the shape of the growth and decline curves. The error bars represent 10 model builds and their respective cross-validation runs. B. Significant days in the random forest classification of the ***Prochlorococcus-Alteromonas*** co-culture curves in E1. The days with the 10 highest feature importance are marked. All curves were aligned to max growth day.

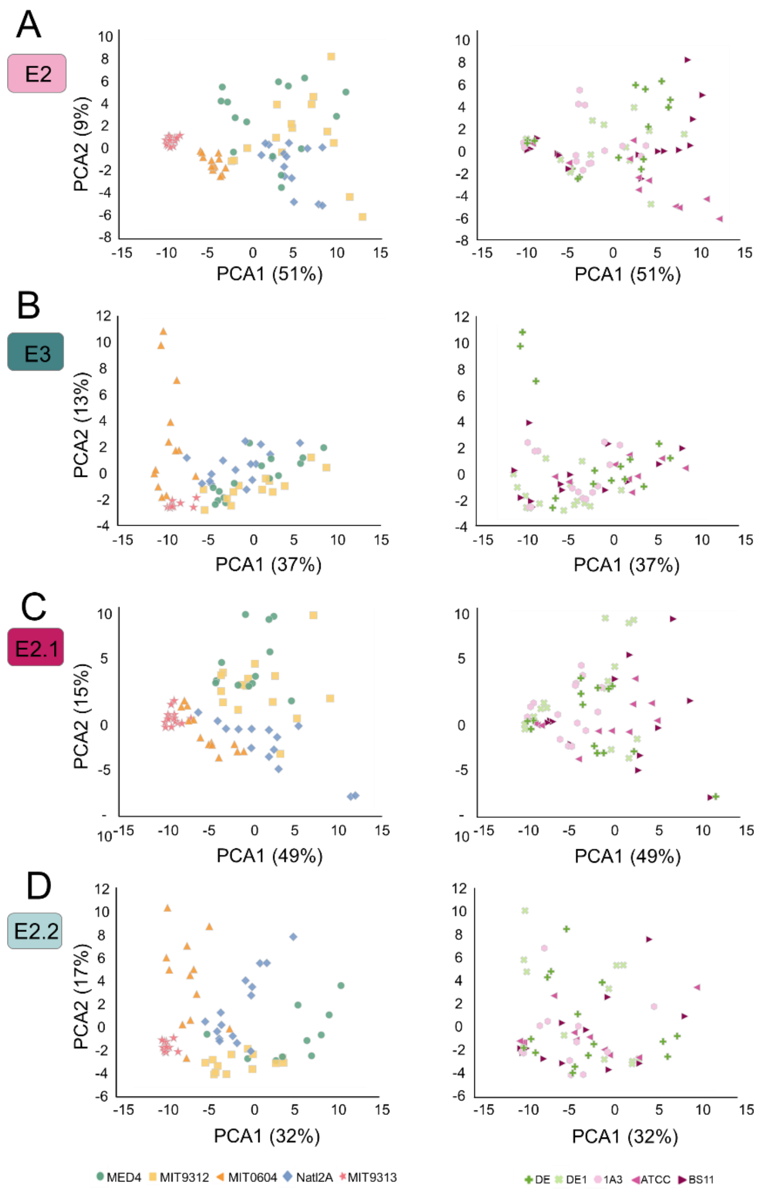

Figure S3: PCA of the fluorescence curves from subsequent experiment. In all experiments, the curve shapes clustered primarily based on the *Prochlorococcus* and only to a much lower extent by the *Alteromonas* strains in co-culture.

Adonis:

A. E2: PRO: $F(4,67) = 13.02$, $R^2 = 0.44$, $p = 0.001$. ALT: $F(4,67) = 3.18$, $R^2 = 0.16$, $p = 0.001$

B. E3: PRO: $F(4,60) = 8.85$, $R^2 = 0.37$, $p = 0.001$. ALT: $F(4,60) = 1.96$, $R^2 = 0.12$, $p = 0.02$

C. E2.1: PRO: $F(4,68) = 14.07$, $R^2 = 0.47$, $p = 0.001$. ALT: $F(4,68) = 2.79$, $R^2 = 0.15$, $p = 0.001$

D. E2.2: PRO: $F(4,59) = 10.08$, $R^2 = 0.41$, $p = 0.001$. ALT: $F(4,59) = 0.72$, $R^2 = 0.05$, $p = 0.8$

PRO: *Prochlorococcus* (left pane), ALT: *Alteromonas* (right pane)

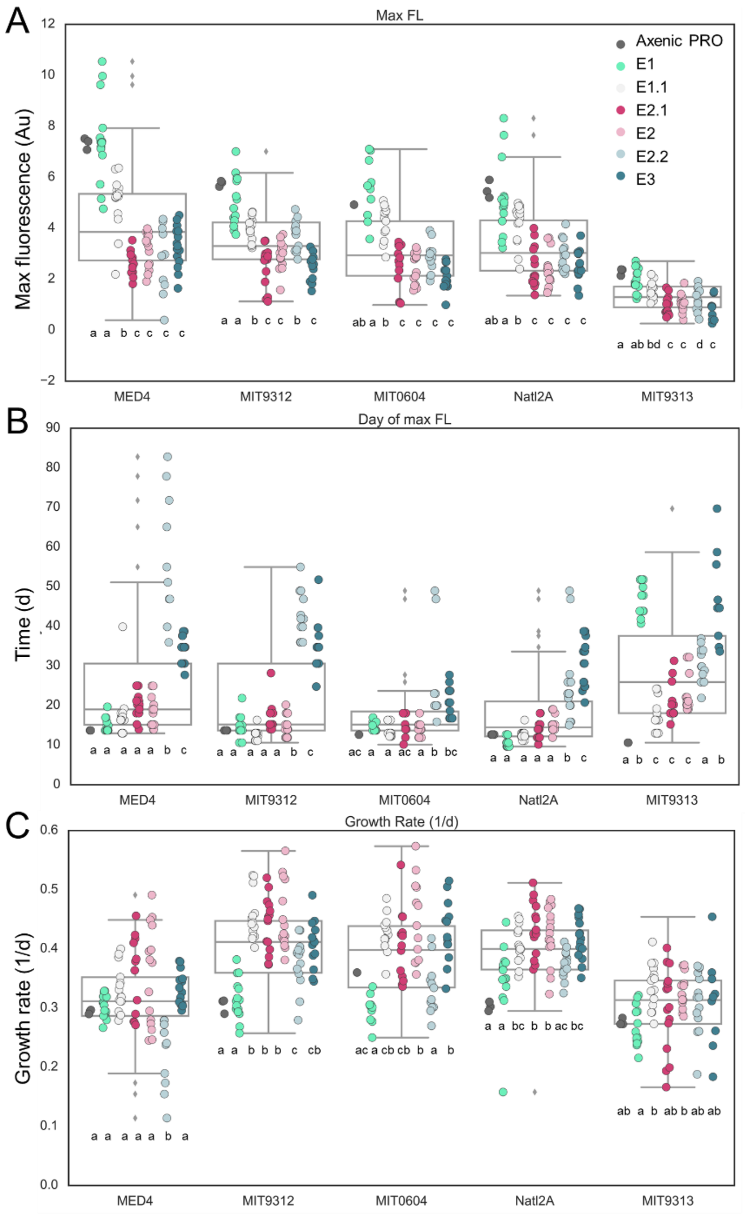

Figure S4: Growth and growth rate of subsequent co-cultures. In all panels, characters indicate results of ANOVA with Bonferroni correction between the different transfers per *Prochlorococcu*s strain (different characters indicate $p < 0.05$). A. Maximum Fluorescence (proxy for number of cells) B. Day of max Fluorescence. C. Growth rates (Axenic PRO: Axenic *Prochlorococcus* in E1). In the subsequent co-cultures (after E1), the growth rate of *Prochlorococcus* increased compared to the first interaction between *Prochlorococcus* and *Alteromonas.* One exception was E2.2, in which growth rates were similar to E1 or (in the case of MED4) even lower. We currently have no explanation for this observation.

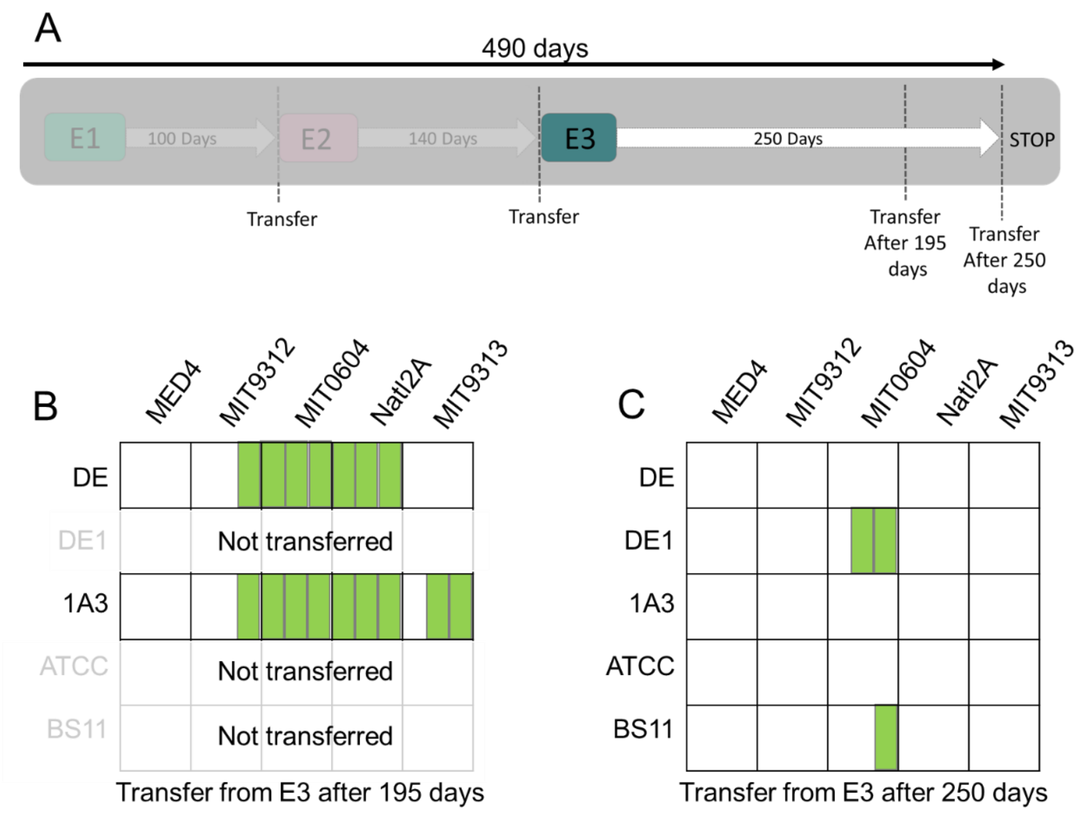

Figure S5: Co-cultures able to survive for extended period. A. Co-cultures from E3 were transferred to new media at two time-points (195 and 250 days). B. On day 195, only co-cultures with *A. macleodii* HOT1A3 and *A. mediterranea* DE were transferred (a total of 30 cultures), of which 16 survived transfer (green squares). C. After 250 days all 75 co- cultures were transferred into fresh media, with only three surviving transfer.

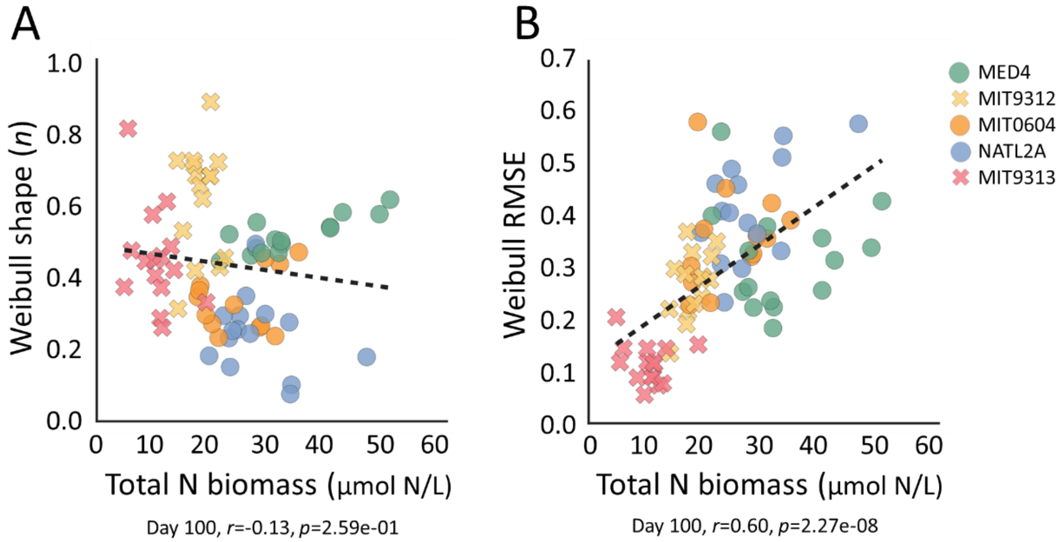

Figure S6: Correlations between total N biomass on day 100, the Weibull shape (A) and the Weibull RMSE (B). Pearson's *r* and *p* values are shown below the plots.

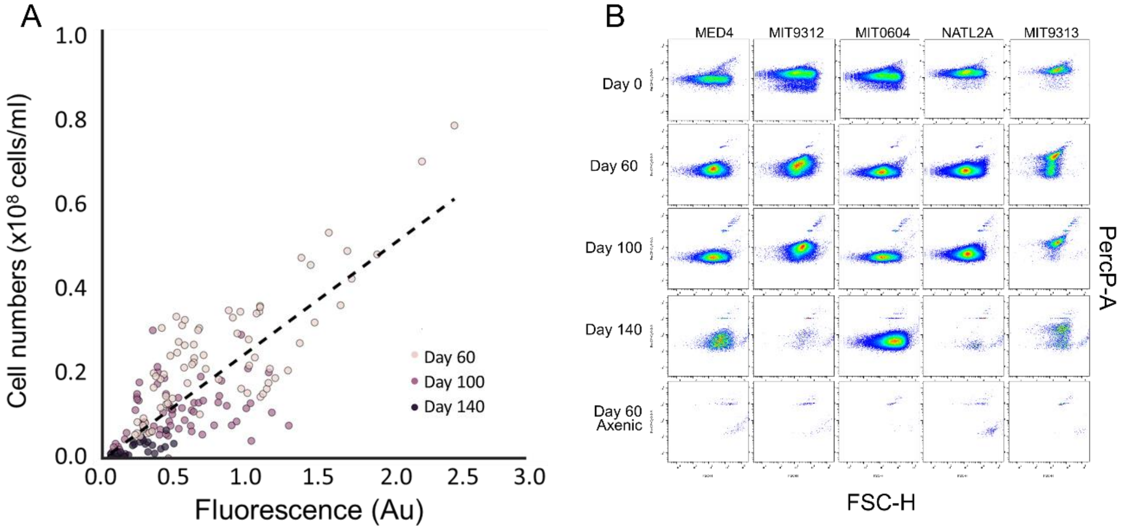

Figure S7: Relationship between cell number and fluorescence during decline. A) Correlation of Fluorescence measurements to Flow cytometry cell numbers. The fit of ordinary least squares is strong, indicating that Fluorescence is a good proxy for cell numbers (OLS $F_{217} = 508$, p = 8e-59, $R^2 = 0.7$). B) Representative Flow cytometry scattergrams of *Prochlorococcus* Co-cultures with *Alteromonas* DE. Co-cultures with other *Alteromonas* strains exhibited similar plots. The x axis is the forward scatter (FSC, a proxy for cell size), and the y axis is the chlorophyll autofluorescence of the cells (PerCP). The per-cell chlorophyll is not completely stable over the growth and decline curves. Firstly, in all strains, the mode of cell fluorescence is lower during long-term starvation compared to exponential growth. Secondly, in MIT9313 (and to a lesser extent in MIT9312 and NATL2A) we observed the emergence of chlorotic sub-populations, in agreement with previous studies [3, 12] However, the actual dynamics are more complex, for instance, in MIT9313 there is a higher fraction of chlorotic cells on days 60 and 140 compared with day 100. Further experiments with a higher temporal resolution are required to determine to what extent these dynamic changes are robust.

SI References

1. Sher D, Thompson JW, Kashtan N, Croal L, Chisholm SW. Response of *Prochlorococcus* ecotypes to co-culture with diverse marine bacteria. *ISME J* 2011; **5**: 1125–1132.
2. Grossowicz M, Roth-Rosenberg D, Aharonovich D, Silverman J, Follows MJ, Sher D. *Prochlorococcus* in the lab and in silico: The importance of representing exudation. *Limnol Oceanogr* 2017; **62**: 818–835.
3. Roth-Rosenberg D, Aharonovich D, Omta AW, Follows MJ, Sher D. Dynamic macromolecular composition and high exudation rates in *Prochlorococcus*. *Limnol Oceanogr* 2021; **66**: 1759–1773.
4. Aharonovich D, Sher D. Transcriptional response of *Prochlorococcus* to co-culture with a marine *Alteromonas*: Differences between strains and the involvement of putative infochemicals. *ISME J* 2016; **10**: 2892–2906.
5. Qi Y. Random Forest for Bioinformatics BT - Ensemble Machine Learning: Methods and Applications. In: Zhang C, Ma Y (eds).2012. Springer US, Boston, MA, pp 307–323.
6. Martiny AC, Ma L, Mouginot C, Chandler JW, Zinser ER. Interactions between thermal acclimation, growth rate, and phylogeny influence *Prochlorococcus* elemental stoichiometry. *PLoS One* 2016; **11**: 1–12.
7. Bertilsson S, Berglund O, Karl DM, Chisholm S. Elemental composition of marine *Prochlorococcus* and *Synechococcus*: Implications for the ecological stoichiometry of the sea. *Limnol Oceanogr* 2003; **48**: 1721–1731.
8. Pedler BE, Aluwihare LI, Azam F. Single bacterial strain capable of significant contribution to carbon cycling in the surface ocean. *Proc Natl Acad Sci U S A* 2014; **111**: 7202–7207.
9. Posacka AM, Semeniuk DM, Maldonado MT. Effects of copper availability on the physiology of marine heterotrophic bacteria. *Front Mar Sci* 2019; **5**: 1–19.
10. Liefer JD, Garg A, Fyfe MH, Irwin AJ, Benner I, Brown CM, et al. The macromolecular basis of phytoplankton C:N:P under nitrogen starvation. *Front Microbiol* 2019; **10**: 1–16.
11. Chan LK, Newton RJ, Sharma S, Smith CB, Rayapati P, Limardo AJ, et al. Transcriptional changes underlying elemental stoichiometry shifts in a marine heterotrophic bacterium. *Front Microbiol* 2012; **3**: 1–24.
12. Roth-Rosenberg D, Aharonovich D, Luzzatto-Knaan T, Vogts A, Zoccarato L, Eigemann F, et al. *Prochlorococcus* cells rely on microbial interactions rather than on chlorotic resting stages to survive long-term nutrient starvation. *mBio* 2020; **11**: 1–13.
13. Pujo-Pay M, Conan P, Oriol L, Cornet-Barthaux V, Falco C, Ghiglione JF, et al. Integrated survey of elemental stoichiometry (C, N, P) from the western to eastern Mediterranean Sea. *Biogeosciences* 2011; **8**: 883–899.
14. Crane SR, Moore JA. Modeling enteric bacterial die-off: A review. *Water, Air, Soil Pollut* 1986; **27**: 411–439.
